# *Mycobacterium avium* Subsp. *avium* and Subsp. *hominissuis* Give Different Cytokine Responses after *in vitro* Stimulation of Human Blood Mononuclear Cells

**DOI:** 10.1371/journal.pone.0034391

**Published:** 2012-04-10

**Authors:** Johanna Thegerström, Bodil Jönsson, Lars Brudin, Björn Olsen, Agnes E. Wold, Jan Ernerudh, Vanda Friman

**Affiliations:** 1 Department of Clinical and Experimental Medicine, Faculty of Health Sciences, Linköping University, Linköping, Sweden; 2 Department of Clinical Physiology, Kalmar County Hospital, Kalmar, Sweden; 3 Department of Infectious Medicine, Institute of Biomedicine, University of Gothenburg, Gothenburg, Sweden; 4 Department of Medicine and Health Sciences, Linköping University, Linköping, Sweden; 5 Infectious Diseases, Department of Medical Sciences, Uppsala University and Academic Hospital, Uppsala, Sweden; 6 Department of Infectious Diseases, Sahlgrenska University Hospital, Gothenburg, Sweden; The Scripps Research Institute Scripps Florida, United States of America

## Abstract

**Background:**

*Mycobacterium avium* is the principal etiologic agent of non-tuberculous lymphadenitis in children. It is also a known pathogen for birds and other animals. Genetic typing of *M. avium* isolates has led to a proposal to expand the set of subspecies to include *M. avium* subsp. *hominissuis*. Isolates associated with disease in humans belong to this subspecies.

**Methodology/Principal Findings:**

Peripheral blood mononuclear cells from six healthy blood donors were stimulated *in vitro* with ten isolates of *M. avium avium* and 11 isolates of *M. avium hominissuis* followed by multiplex bead array quantification of cytokines in supernatants. *M. avium hominissuis* isolates induced significantly more IL-10 and significantly less IL-12p70, TNF, IFN-γ and IL-17 when compared to *M. avium avium* isolates. All strains induced high levels of IL-17, but had very low levels of IL-12p70.

**Conclusion/Significance:**

The strong association between *M. avium* subsp. *hominissuis* and disease in humans and the clear differences in the human immune response to *M. avium* subsp. *hominissuis* compared to *M. avium* subsp. *avium* isolates, as demonstrated in this study, suggest that genetic differences between *M. avium* isolates play an important role in the pathogenicity in humans.

## Introduction

The *Mycobacterium avium* complex (MAC), consisting of *Mycobacterium avium* and *intracellulare*, was in the 1950s reported as opportunistic bacteria causing disease in humans [1]. With the emergence of the AIDS epidemic *M. avium* gained further attention as it caused disseminated disease among immunocompromised patients. MAC also causes pulmonary disease, mainly in elderly people [2]. Chronic cervical adenitis caused by MAC may be seen in otherwise healthy toddlers. These children usually present a painless enlarged lymph node in the neck region. If the affected lymph nodes are not surgically removed then cold abscesses and spontaneous fistulas may develop [3]. Histopathology typically reveals granulomatous necrotic inflammation with epiteloid cells and polymorphonuclear neutrophils in the necrotic central area [4].

Non-tuberculous mycobacteria (NTM) are ubiquitous in the environment, particularly in water [5]. *M. avium* is comprised of the subspecies *M. avium avium*, *M. avium silvaticum* and *M. avium paratuberculosis*. Commonly, *M. avium avium* strains are differentiated from other *M. avium* subspecies using the method of restriction fragment length polymorphism (RFLP) for the insertion sequences IS*1245* and IS*901*. *M. avium avium* strains exhibit a typical three-band, “bird-type” pattern while the proposed new subspecies *M. avium hominissuis* strains (usually human/porcine origin) generate multiple bands on RFLP for the insertion sequence IS*1245* [6]. The majority of human clinical isolates are of the *M. avium hominissuis* subspecies, which, in addition to pigs, is occasionally isolated from captive birds. In contrast, *M. avium avium* is rarely isolated from man, and has not been found in children with *M. avium* lymphadenopathy [7], [8]. Instead it is isolated from birds and a variety of mammals of both wild and domestic species.

In a host, *M. avium* causes an intra-cellular infection which primarily affects monocytes and macrophages. A T helper 1 (Th1) response normally increases macrophage bactericidal capacity. The importance of Th1 responses for clearance of mycobacteria is demonstrated by various mutations affecting the IL-12/IFN-γ axis leading to susceptibility to normally avirulent mycobacterial species [9], [10]. Tumour necrosis factor (TNF) and IL-12 are important cytokines early in infection with IL-12 being responsible for the induction of antigen-specific CD4+ cells which produce the Th1-signature cytokine IFN-γ [11], [12]. Another T helper cell subpopulation, named Th17 because of its IL-17 production, has been ascribed an important role in the innate and acquired immunity against infections [13], including tuberculosis [14]. Its function in protection against environmental mycobacteria has not been determined. IL-10 is an immunoregulatory cytokine which down-regulates Th1 activation. This benefits the host when it limits the inflammatory response and ensuing tissue damage. Inappropriate expression of this cytokine may however weaken the immune response to a point where it leads to worsened or uncontrolled infection [15].

There is evidence, both by sensitin skin test studies [16] and studies of antibody levels against mycobacterial antigens in different age groups [17], that a vast majority of the population comes in contact with MAC bacteria early in life. Nevertheless, only about 5/100,000 children develop disease [18], and furthermore, are never infected with the bird-type strains. The reason for the paucity of *M. avium avium* infections among humans is not known. In theory, more than one explanation is possible: the “bird-type” strains might proliferate exclusively in special environmental niches, thereby being exposed to animals but not to humans. Alternatively, phenotypic differences between the bacterial strains could render some strains unable to infect humans. Exploring this hypothesis we examined differences in the immune response to two distinct genetic classes of *M. avium* isolates. We therefore selected ten isolates of *M. avium avium* and eleven isolates of *M. avium hominissuis* that we had previously characterized using RFLP IS *1245*. We then compared the ability of these isolates to induce cytokine responses by *in vitro* stimulation of human peripheral blood mononuclear cells (PBMCs) from healthy blood donors.

## Methods

### Ethics statement

Blood samples were obtained from healthy blood donors who had given their informed consent in writing (under a protocol developed by the Department of Transfusion Medicine, Blood bank, Sahlgrenska University Hospital in compliance with the Swedish Board for Accreditation and Conformity Assessment's (Swedac) Standard SS-EN ISO 15189).

### Strains and restriction fragment length polymorphism (RFLP)

*M. avium* isolates from different sources (Table 1) which were isolated between 1983 and 2002 were obtained from either the Swedish Institute for Infectious Disease Control (SMI) or one of the clinical microbiology laboratories in Sweden, namely at Karolinska University Hospital in Stockholm, Sahlgrenska University Hospital in Gothenburg, Linköping University Hospital or Umeå University Hospital.

**Table 1:** *Mycobacterium avium* strains included in this study.

Isolate nr	Animal species	Isolation site	RFLP profile	*M. avium* subspecies
1	Pig	Liver	3-band	*M. avium avium*
2	Pig	Liver	3-band	*M. avium avium*
3	Pig	Liver+Lymph node	3-band	*M. avium avium*
4	Pig	Liver	3-band	*M. avium avium*
5	Pheasant	Granuloma	3-band	*M. avium avium*
6	Buzzard	Liver	3-band	*M. avium avium*
7	Cow	Lymph node	3-band	*M. avium avium*
8	Horse	?	3-band	*M. avium avium*
9	Cat	?	3-band	*M. avium avium*
10	Human (adult)	Sputum	3-band	*M. avium avium*
11	Human (child)	Lymph node	Multiband	*M. avium hominissuis*
12	Human (child)	Lymph node	Multiband	*M. avium hominissuis*
13	Human (child)	Lymph node	Multiband	*M. avium hominissuis*
14	Human (child)	Lymph node	Multiband	*M. avium hominissuis*
15	Human (child)	Lymph node	Multiband	*M. avium hominissuis*
16	Human (child)	Lymph node	Multiband	*M. avium hominissuis*
17	Human (child)	Lymph node	Multiband	*M. avium hominissuis*
18	Human (child)	Lymph node	Multiband	*M. avium hominissuis*
19	Human (child)	Lymph node	Multiband	*M. avium hominissuis*
20	Human (child)	Lymph node	Multiband	*M. avium hominissuis*
21	Human (child)	Lymph node	Multiband	*M. avium hominissuis*

The isolates had previously been characterized by RFLP for IS*1245* [8] and as reported by Thegerström [Abstract, Seventh International Conference on the Pathogenesis of Mycobacterial Infections, 2008, Saltsjöbaden, Sweden]. Ten isolates showing the typical “bird-type”, three-band profile on RFLP IS*1245* were chosen to represent as many different hosts as possible, including the only isolate of this type from a human (in sputum of an HIV-negative adult where the clinical relevance was unknown). Eleven isolates from the lymph nodes of children were selected to represent different subgroups of RFLP IS*1245* multiband profiles.

For non-mycobacterial controls *Streptococcus mitis*, isolated from human oral cavity, was selected as a representative of a typical Gram-positive bacterium. *Escherichia coli*, isolated from human urine (cystitis), was used as a representative of a typical Gram-negative bacterium. Both originated from the Culture Collection of the University of Gothenburg, Department of Infectious Medicine, Sweden [19].

### Preparation of bacteria for stimulation tests

Mycobacterial isolates were cultured on Middlebrook 7H10 agar for four weeks, then harvested in Dubecco's endotoxin-free PBS (PAA laboratories, GmbH, Pasching, Austria) and vortexed vigorously to obtain a homogenous suspension. After sedimentation for 15 min to remove lumps, bacterial suspensions were transferred to six-well tissue culture plates and UV-irradiated for 1 h. *S. mitis* and *E. coli* were cultured aerobically on horse blood agar plates (Bacteriological laboratories, Sahlgrenska University Hospital, Gothenburg, Sweden) at 37°C for 24 h and UV-irradiated for 15–18 min. Sterilization was confirmed by a zero viable count after eight weeks on Middlebrook agar (Bacteriological laboratories, Sahlgrenska University Hospital) for the mycobacteria and after 48 h on horse blood agar for the control strains.

Inactivated bacteria were counted in a Bürker chamber and the concentration adjusted to 5×10^8^ bacteria/mL and stored at −70°C until used.

### Isolation and stimulation of PBMC

Mononuclear cells were isolated from blood donor buffy coats by density gradient centrifugation (820× g, room temperature, 20 min, Lymphoprep, Nyegaard, Norway), washed ×3 (460× g, 10 min) in ice-cold endotoxin-free RPMI 1640 medium with 2 mM L-glutamine (Invitrogen, Carlsbad, CA) and resuspended at 2×10^6^ cells/mL in complete RPMI medium (supplemented with 0.01% gentamicin (Sigma, St. Louis, MO) and 5% inactivated endotoxin-free foetal calf serum (Invitrogen)). PBMC suspensions were aliquoted (200 µL) in flat-bottomed 96-well microtiter plates (Nunc, Roskilde, Denmark) and bacterial suspension was added to achieve a final bacterial concentration of 5×10^6^ or 5×10^7^ bacteria/mL, corresponding to 2.5 and 25 bacteria per mononuclear cell. *S. mitis* was used at 5×10^6^ and *E. coli* at 5×10^7^ based on previous studies of optimal responses [19].

The cultures were incubated at 37°C in a humidified atmosphere supplemented with 5% CO_2_. Supernatants were harvested at 24 h for measurement of IL-10, IL-12p70 and TNF and after five days for IFN-γ and IL-17. Supernatants were frozen at −70°C until analyzed.

### Blocking of IL-10

Anti-human IL-10 antibodies (JES3-19F1, from BioLegend, San Diego, CA) were pre-incubated with cell cultures at concentrations of 0, 1 and 5 µg/mL for 45 min at 4°C, prior to the addition of 5×10^6^ bacteria/mL.

### Cytokine measurement by multiplex bead array analysis

The levels of IL-10, IL-12p70, TNF, IFN-γ and IL-17 were analysed in cell culture supernatants according to instructions from the manufacturer (Human Cytokine LINCOplex Kit, LINCO Research, St. Charles, USA) using the Luminex 100 instrument (Luminex Corporation, Austin, Texas, USA) and StarStation software (Applied Cytometry Systems, Sheffield, UK).

Controls (Human Cytokine Controls I and II) and standards were prepared according to the manufacturer's instructions. The coefficient of variation was found to be 7%–24% for Control 1 (mean value 77–93 pg/mL for the included cytokines) and 6%–27% for Control 2 (mean value 911–1205 pg/mL for the included cytokines). The Human Cytokine Standard Cocktail was diluted with Assay Buffer to concentrations of 10 µg/mL, 2 µg/mL, 400 pg/mL, 80 pg/mL, 16 pg/mL, 3.2 pg/mL and 0.64 pg/mL.

### Statistics

After logarithmic transformation cytokine levels were reasonably well normally distributed to allow parametric statistical analyses. Differences between groups were tested by ANOVA, repeated ANOVA followed by Duncan's test and factorial ANOVA when appropriate. P<0.05 was considered significant, and 95% confidence intervals are shown in the figures.

## Results and Discussion

**Figure 1:**
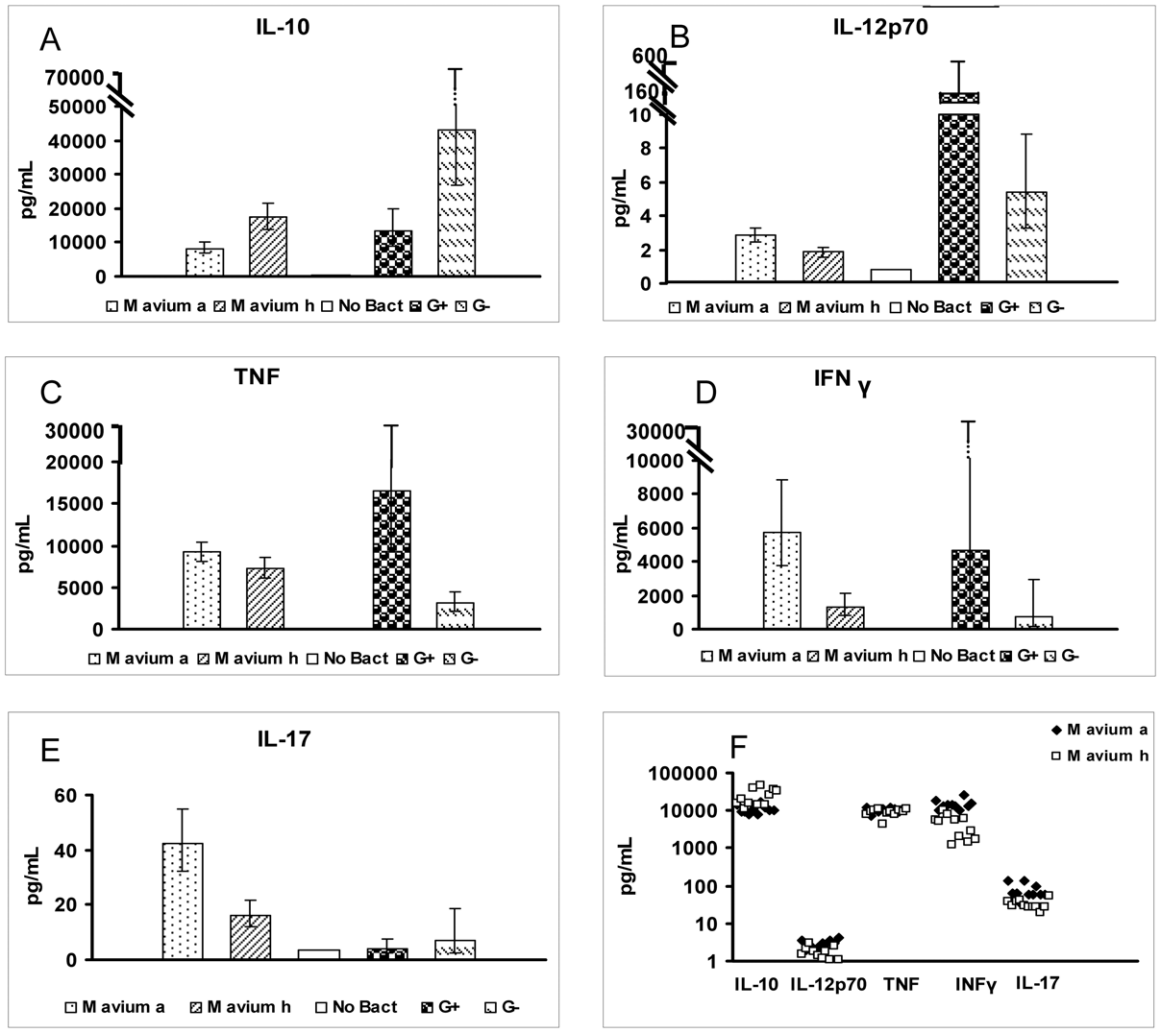
Geometric means of cytokine levels: (A) IL-10, (B) IL-12p70, (C) TNF, (D) IFN-γ, (E) IL-17. Cytokine levels in pg/mL after stimulation with 5×10^7^ inactivated bacteria/mL of PBMCs from six healthy blood donors are shown. IL-10, IL-12p70 and TNF were measured after a 24 h stimulation; IFN-γ and IL-17 after five days of stimulation. Geometric means and 95% confidence intervals are shown. F) **Arithmetic means of cytokine levels.** The arithmetic mean of the cytokine level (pg/mL) for each individual isolate is shown. M avium a = *Mycobacterium avium avium*; M avium h = *Mycobacterium avium homunissuis*; No Bact = no bacteria; G+ = *Streptococcus mitis*; G− = *Escherichia coli*. Note that Figure F is shown on a logarithmic scale.

Blood donor PBMCs were stimulated with 21 strains of inactivated mycobacteria and Gram-positive/Gram-negative control bacteria. *M. avium hominissuis* strains (n = 11) induced significantly more IL-10 (p<0.001) than did *M. avium avium* strains (n = 10) and, conversely, less IL-12p70 (p = 0.0015), TNF (p = 0.029), IFN-γ (p<0.001) and IL-17 (p<0.001) at a bacterial concentration of 5×10^7^ inactivated bacteria/mL (Figure 1 A–F).

IFN- γ seems to be the key cytokine in the defence against mycobacteria [20]. The substantial difference in the levels of IFN- γ (300%) induced by *M. avium hominissuis* and *M. avium avium* leads us to hypothesize that the higher levels of IFN-γ induced by *M. avium avium* might prevent infection by this subspecies whereas the lower levels induced by *M. avium hominissuis* may permit this subspecies to infect humans, for example causing lymphadenitis in children.

IL-12 has been considered to have a major role in the defence against *M. avium* primarily through activating a Th1 response, including IFN- γ-producing T cells and NK cells [12].

**Figure 2:**
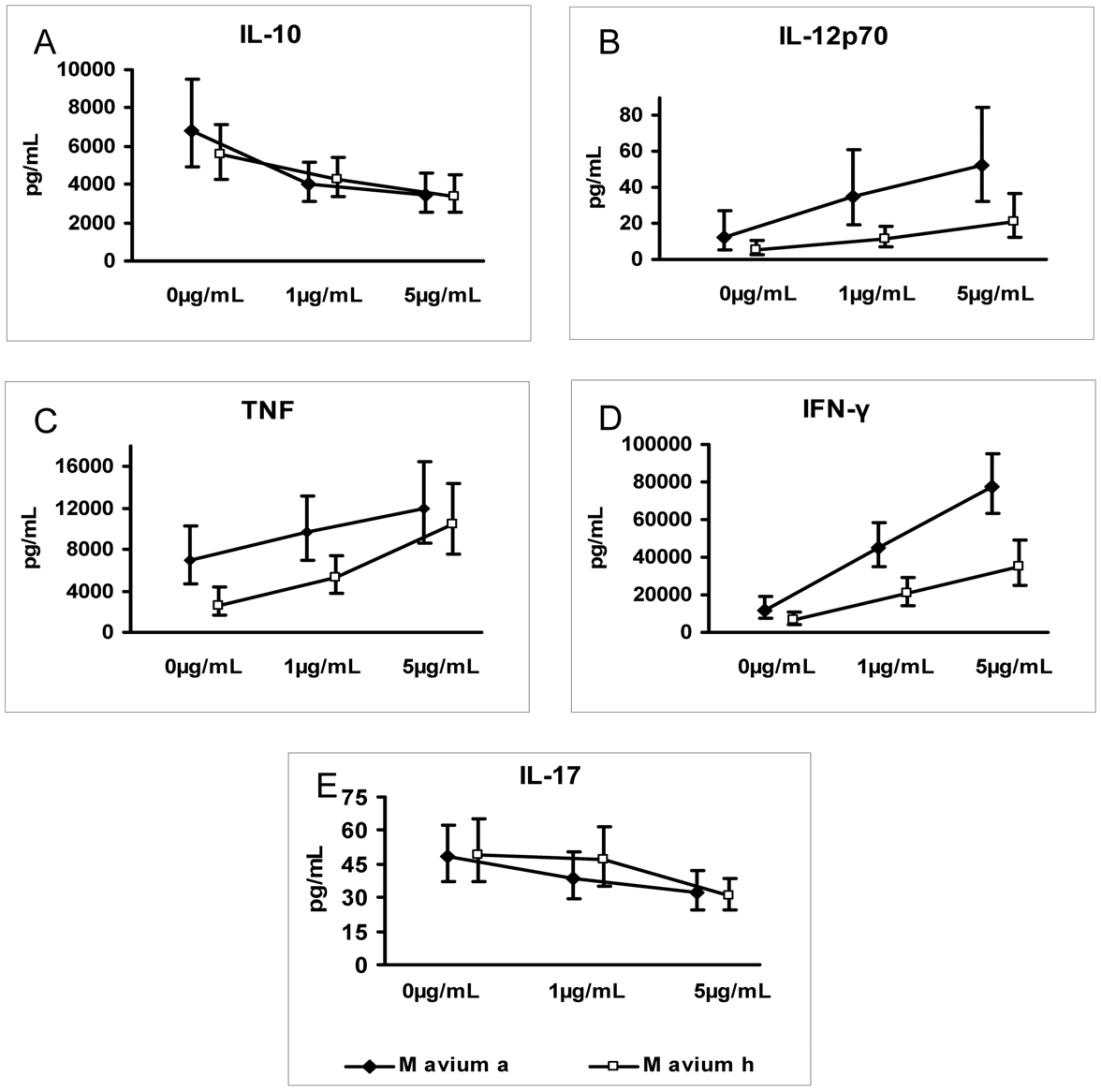
Cytokine levels when blocking IL-10: (A) IL-10, (B) IL-12p70, (C) TNF, (D) IFN-γ, (E) IL-17. Cytokine levels in pg/mL after stimulation with 5×10^6^ inactivated bacteria/mL of PBMCs from four healthy blood donors that had been pre-incubated with anti-IL-10 antibodies at 0, 1 and 5 µg/mL respectively are shown. M avium a = *Mycobacterium avium avium*; M avium h = *Mycobacterium avium homunissuis*.

The production of IL-12p70 in response to mycobacterial preparations in our study was very low, similar to the response to *E. coli* (Figure 1 B). We hypothesized that the capacity of the mycobacterial preparations to induce IL-12p70 was being masked by their more potent induction of IL-10. This particular cytokine has an immunoregulatory function and is known to inhibit, for example IL-12 and TNF [15]. We investigated this hypothesis by adding neutralizing antibodies to IL-10 (Figure 2 A–E). The stimulations in this experiment were done with mycobacterial preparations at a lower bacterial concentration (5×10^6^ inactivated bacteria/mL); which likely accounts for the discrepancy at baseline (0 µg/mL) when compared with the results shown in Figure 1. When IL-10 was blocked, levels of IL-12p70, TNF and IFN-γ increased 2–5 times (p<0.001) in response to both *M. avium avium* and *M. avium hominissuis*. However, the levels of IL-12 were still low compared to the Gram-positive control. We had previously found that several environmental mycobacteria, including *M. avium*, are very poor inducers of IL-12 (Jönsson B et al., manuscript in preparation). This trait may allow the mycobacteria to persist within phagocytes by avoiding triggering bactericidal mechanisms. In the absence of IL-10, significant differences in the levels of IFN-γ and IL-12p70 were still found when PBMCs were stimulated by either *M. avium hominissuis* or *M. avium avium*. This shows that the difference between the groups could not be ascribed to their different ability to induce IL-10 alone.

More recently, it has been speculated that IL-17 plays a role in protection against mycobacterial infection [21], [22]. Supporting this, we found that both subtypes of *M. avium* are potent inducers of IL-17, *M. avium avium* to a greater extent than *M. avium hominissuis* (a 160% difference). In parallel with the reasoning concerning Th1 and IFN- γ, a weaker Th17 response might also explain why *M. avium hominissuis* is more prone to infect humans than *M. avium avium*.

Children with clinical disease by *M. avium* constitute only a small proportion of the exposed children who never develop disease, judging by sensitin skin test studies [16]. Specific immune deficiencies in the host are thought to explain the development of clinical disease in certain children. Children with the now well known hereditary conditions of Mendelian susceptibility to poorly virulent mycobacterial species [9], [10], with mutations affecting the IL-12/IFN- γ/STAT1-axis, have severe or recurrent diseases of *M. avium* infections. Similarly, mutations in the IL-23/IL-17-axis could also be well worth investigating in children with *M. avium* chronic lymphadenitis as a low IL-17 response early in the course of infection in some individuals might allow the bacteria to establish themselves.

IL-17 is also a cytokine that seems to be important in the pathogenesis of autoimmune disease, in inflammation in general and in tuberculosis mediated immunopathology in particular [22], [23]. We speculate that the large amounts of IL-17 induced by both groups of *M. avium* (compared to both Gram positive and Gram negative controls, Figure 1 E) are likely to play a significant role in the pathogenesis of mycobacterial disease in humans. Considering the histopathology of *M. avium* lymphadenitis, several features can be identified that are likely to be caused by elevated IL-17 levels. Interestingly, there are two different types of granuloma formation in nontuberculous mycobacterial disease. Children with Mendelian susceptibility to poorly virulent mycobacteria display “lepromatous-like” granulomas (type II) containing vast amounts of macrophages loaded with high numbers of mycobacteria while affected children without this immunodeficiency display well-defined “tuberculoid” granulomas (type I) [24]. These granulomas are characterized by the presence of neutrophils scattered throughout the necrotic foci (IL-17 recruits neutrophils to inflammatory sites [25]) and frequent observations of Langhans' giant cells (IL-17 stimulates fusion of dendritic cells with formation of giant cells [26]). As acid fast bacilli are normally scant in number, it may be difficult to even obtain a positive culture for diagnosis. The low number of bacteria suggests that the majority of children with *M. avium* lymphadenitis are not necessarily incapable of eliminating the mycobacteria. Local inflammation and tissue destruction mediated by IL-17 might instead be the greater problem in the pathogenicity of this disease.

The levels of IL-17 are probably dependant on IFN-γ levels as IFN-γ has been shown to inhibit IL-17 production *in vitro* in response to BCG infection [25]. Our results indicate that this is also true for *M. avium* since IL-17 levels decreased (p<0.001) while IFN-γ increased in the absence of IL-10 (Figure 2). This suggests that *in vivo*, in mycobacterial disease, IL-17 might not be sufficiently down-regulated since IFN-γ levels have been shown to decrease in the late course of infection [27].

We speculate that as high levels of IL-17 early on in an infection might be beneficent to the host (and contribute to explain why humans get rid of *M. avium avium* but not *M. avium hominissuis*), high levels later on in an infection might lead to an inflammatory response which causes tissue damage in a similar manner to the immunopathology which occurs in association with a chronic *M. tuberculosis* infection [23].

In this study we find a highly significant difference in the human immune response to two genetically distinct *M. avium* subspecies. Different glycopeptidolipids (GPLs) expressed on the surface of the mycobacteria might be the phenotypic feature accounting for the different effects on the immune system. Interestingly, it has been reported that the innate immune system response is activated by the binding of GPLs to the Toll-like cell receptor 2 (TLR2) [28]. Mycobacterial surface lipid extracts have been shown to elicit large quantities of IL-17 in similar stimulation studies as ours (Jönsson B et al, in manuscript). Other phenotypic features of the mycobacteria might however be of importance as well, since there is no consistent correlation between serotypes (based on the highly antigenic GPLs) and the respective subspecies [6], [29].

Taking our results into consideration, the strong association between *M. avium hominissuis* and disease in humans would be explained by specific interactions between this particular subspecies and the human immune system. It is likely that the immune system of other mammals and birds interact differently with these mycobacteria as many mammals, and birds in particular, are equally or more prone to infections with the genetically distinct subspecies *M. avium avium*.

The genetic diversity of the MAC has not been recognized until recently. *M. avium hominissuis* is a proposed name but has not been formally accepted yet [30]. As such, earlier studies on strains that have not been classified into one of the main genetic groups of MAC should be treated with caution. The results of this study underscore the importance of characterizing the *M. avium* strains used in immunological studies as either *M. avium avium* or *M. avium hominissuis*.

In conclusion we here demonstrate that two genetically distinct groups of *M. avium*, namely the proposed subspecies *M. avium hominissuis* and *M. avium avium*, induce different cytokine secretion in human PBMCs. The higher secretion of suppressive IL-10, together with the lower secretion of anti-microbial Th1- and Th-17-associated cytokines by *M. avium hominissuis* compared to *M. avium avium*, are likely to explain why humans are almost exclusively infected by *M. avium hominissuis* and not by *M. avium avium*.
